# Phytoremediation: Sustainable Solutions for Heavy Metal Pollution and Bioenergy in Bangladesh

**DOI:** 10.1155/tswj/5510989

**Published:** 2025-05-18

**Authors:** Md Nasir Ahmed, Chowdhury Alfi Afroze, Rownak Jahan

**Affiliations:** ^1^Department of Biotechnology & Genetic Engineering, University of Development Alternative, Dhaka, Bangladesh; ^2^Department of Environmental Science and Management, Independent University, Dhaka, Bangladesh

**Keywords:** biomass energy, bioremediation, green technology, phytoremediation, phytotechnology

## Abstract

Heavy metal pollution is plundering the well-being of people in Bangladesh, a developing country in South Asia, which is also striving to secure green energy resources. This comprehensive review highlights the use of phytoremediation technology to both clean up heavy metal pollution and produce bioenergy in Bangladesh. It emphasizes the dual benefits of removing contaminants from the environment while generating sustainable energy sources. We identified 16 species of hyperaccumulators with potential as bioenergy sources, including second-generation bioethanol, biodiesel, biofuels, biogas, and bioelectricity. This effort supports the adaptability of Sustainable Development Goal 7—access to affordable, reliable, sustainable, and modern energy for all—and is aimed at helping Bangladesh achieve its target of generating 15% of total electricity from renewable sources by 2041. These species were effective in accumulating heavy metals from contaminated soils and wastewater. Bioenergy plants with hyperaccumulating activities can address the issue of biomass disposal by converting bioenergy production after the remediation process of chemical pollutants, leading to bioeconomic technology and increasing the acceptance of large-scale applications. Using advanced biotechnological approaches, it is possible to improve hyperaccumulating activities such as absorbing, accumulating, biodegrading, or immobilizing heavy metals. The review further provides recommendations, including funding initiatives, public awareness campaigns, collaborative efforts, policy development, and capacity building to enhance the development and implementation of phytoremediation technology using bioenergetic plants in Bangladesh, thereby unlocking its bioeconomic potential.

## 1. Introduction

Improper management of pollution, including industrial, medical, and agricultural toxins, as well as the rise of hazardous and toxic chemicals, poses an emergency threat to public health, the ecosystem, biodiversity, and agriculture. The Earth has become a “toxic planet” for humanity as a result of this silent threat of pollution [1]. Pollution is responsible for approximately nine million premature deaths per year worldwide, with a higher death rate in low- and middle-income countries than in high-income countries [2].

Bangladesh is currently facing a serious problem with chemical pollutants, especially heavy metals such as arsenic, cadmium, chromium, and lead. These pollutants can be found in food, soil, and water due to municipal and industrial wastewater, underground contamination, tannery, textile, and pharmaceutical effluents, chemical fertilizers, and pesticides [3, 4]. Heavy metal contamination of surface and groundwater is particularly alarming, with 83.3% of water bodies exceeding the standard threshold limit of 100 on the heavy metal pollution index, making it unsafe for consumption [5, 6]. Heavy metal pollution is widespread in Bangladesh, including coastal locations [7], and almost everything is affected. For instance, foods [8, 9], soil [10, 11], agricultural land [12, 13], rivers [14, 15], and air [16, 17] are contaminated mainly by arsenic, lead, cadmium, chromium, nickel, and copper. This is a major concern because, between 2005 and 2018, 24,000 premature deaths occurred in Dhaka, Bangladesh, which is the 15th most polluted city in the world [18].

Bioaccumulative phytoremediation technology is a way to remove, degrade, or detoxify heavy metals from the environment [19]. Phytoremediation is a sustainable green technology that uses hyperaccumulator species, which can grow in heavy metal-contaminated soil and water through the concentration of metals in their tissues. Hyperaccumulator plants can accumulate and tolerate large amounts of toxic heavy metals from soil and water, and phytoremediation technology has been emerging around the world due to biotechnological advancements [20, 21]. The term “phytoremediation” was first introduced in 1983 by Chaney [22] and later used by Ilya Raskin of Rutgers University in a 1991 grant proposal [23]. Since then, scientists have advanced the technology of phytoremediation by incorporating relevant omics approaches such as genetic engineering and transgene to improve the efficiency of the accumulation process and the tolerance capability of plants among chemical contaminants [24, 25]. Other environmental cleanup technologies include bioremediation, which utilises microorganisms to sustainably degrade organic contaminants. Nanotechnology presents innovative solutions through the use of nanoparticles as efficient sorbents or catalysts. For water remediation, advanced oxidation processes (AOPs) such as photocatalysis and ozonation effectively degrade recalcitrant pollutants, thereby enhancing water quality. Biochar and other sorbents offer viable options for pollutant removal, while membrane technologies like reverse osmosis provide selective purification for diverse applications [26]. Among these technologies, phytoremediation stands out as a cost-effective and environmentally friendly method, particularly well-suited for addressing large areas of contamination.

Phytoremediation includes four processes: phytoextraction, where pollutants are concentrated in aboveground areas that can be harvested; rhizofiltration, where pollutants are absorbed, concentrated, and/or precipitated from contaminated wastewater; phytostabilization, which prevents the migration of pollutants; and phytovolatization, where pollutants are accumulated and evaporated from the leaf surface as they are converted to volatile forms within the plant [27]. The use of phytoremediation as a commercial phytotechnology is still ongoing, particularly in developing countries such as Bangladesh, where chemical pollution, especially heavy metals, is severely catastrophic. However, this phytotechnology may have significant promise in addressing the emerging problem of heavy metal pollution and enhancing sustainability without giving up continued development.

This paper compiles hyperaccumulators investigated in the lab and native to Bangladesh, selecting plant species with commercially valuable byproducts for bioenergy resources like bioethanol, biodiesel, and bioelectricity. It discusses current technological approaches for plant-based remediation, aiming for field application, and addresses commercialisation limitations. The review emphasizes integrating biomass from phytoremediation of toxic metals into bioenergy production to enhance yield while minimizing pollutant transfer. Overall, our review is aimed at unlocking the bioeconomic potential of phytoremediation and bioenergy production in Bangladesh.

## 2. Literature Search Methodology

We conducted a literature search to gather relevant articles on hyperaccumulators using native plants of Bangladesh. The search included databases such as Google Scholar, PubMed, Springer Nature, Elsevier, and Wiley Online Library. Key search terms included: “Phytoremediation and Bangladesh,” “phytoremediation of toxic metals and Bangladesh,” “plant-assisted heavy metals removal and Bangladesh,” “hyperaccumulators, bioaccumulation and Bangladesh,” and “plant-based remediation of heavy metals and Bangladesh.” There was no specific publication years selected during the search, and articles on hyperaccumulator studies not relevant to Bangladesh were excluded from this study.

## 3. Results

A total of 29 articles have been identified for this study. The total number of phytoremediators is 36 (shown in as Table S1). All of these hyperaccumulator species are native to Bangladesh. According to the lab studies, the selected species were highly efficient for accumulating heavy metals from contaminated soil and wastewater, confirmed by assessing the bioconcentration factor (BCF) (predicting the ratio of heavy metal concentration in the plant tissues) and translocation factor (TF) (phytoextraction ability of a plant to translocate the heavy metals from roots to shoots and leaves). Both are important in the scientific evaluation to determine the potential of the plant for phytoremediation ability. Plants having greater than one (< 1) BCF and TF may be appropriate for heavy metals phytoremediation at a field scale [15]. The calculations of BCF and TF are performed as follows:

i. *BCF*_*leaf*_ = *C*_*leaf*_/*C*_*soil* or *water*_
ii. *BCF*_*stem*_ = *C*_*stem*_/*C*_*soil* or *water*_
iii. TF = BCF of target organ, such as leaf or stem/BCF of root

where *C*_soil⁣or⁣water_ represents the metal concentration in the soil or water. *C*_leaf_ represents the metal concentration in the plant leaf. *C*_stem_ represents the metal concentration in the plant stem.

Various hyperaccumulator species in Bangladesh have shown promise for phytoremediation of heavy metals from different contaminated sources. *Acanthus ilicifolius* L. is effective in accumulating Cu, Zn, As, and Fe from shipbreaking areas in Sitakunda, translocating metals to both roots and shoots [28]. *Ageratum conyzoides* L. efficiently accumulates arsenic from contaminated underground water in various districts, translocating metals to roots and all parts of the plant [29]. *Corchorus capsularis* L. is capable of accumulating arsenic and lead from contaminated soils in Mymensingh and Bhaluka, translocating metals to roots and shoots [30]. *Bryophyllum pinnatum* (Lam.) Oken is effective in accumulating Cu and Zn from textile industry areas, translocating metals to roots, stems, and leaves [31]. *Brassica juncea* (L.) Czern. accumulates multiple heavy metals (Cd, Ar, Cr, Pb, Cu, and Zn) from Buriganga riverside and industrial sediments, translocating metals to roots, shoots, leaves, and fruits [32]. *Xanthium strumarium* Lour. is capable of accumulating Cr from wastewater discharge channels, translocating metals to roots [33]. *Pteris vittata* L. accumulates arsenic from contaminated soils, translocating metals from roots to shoots [34]. *Pennisetum purpureum* Schumach. is effective in accumulating Cr, Zn, Cu, and Pb from tannery sludge, translocating metals to roots, shoots, and leaves [35]. *Spirodela polyrhiza* (L.) Schleid. efficiently accumulates arsenic from test concentrations of arsenate and dimethylarsinic acid, translocating metals to roots [36]. *Chrysopogon zizanioides* (L.) Roberty accumulates arsenic from contaminated soil, translocating metals to roots and leaves [37]. *Ipomoea carnea* Jacq. is capable of accumulating Cr and As from contaminated soil [38]. *Eichhornia crassipes* (Mart.) Solms is efficient in remediating arsenic, Cr, and Ni from underground water and pharmaceutical wastewater [39]. *Azolla caroliniana* Willd. is effective in remediating eutrophic water and improving physiochemical properties from polluted lakes in Dhaka City [40]. *Pistia stratiotes* L. is capable of remediating eutrophic water and pharmaceutical wastewater, accumulating Cr and Ni [39, 41]. *Schumannianthus dichotomus* (Kuntze) Veldkamp & IM Turner is efficient in treating biochemical pollutants in lab-scale vertical subsurface flow constructed wetlands [42]. These findings highlight the potential of various hyperaccumulator species for effective phytoremediation of heavy metals from different contaminated sources in Bangladesh.

### 3.1. Exploring the Bioenergy Potential of Native Hyperaccumulator Species

This ongoing study has identified a total of 20 native hyperaccumulators from Bangladesh, which have been investigated under laboratory or greenhouse conditions. Furthermore, the biomass of these hyperaccumulator species has the potential for bioeconomically valuable resources, as shown in Table 1. Additionally, this study identified 16 native hyperaccumulator species in Bangladesh that show promise for producing biogas, biodiesel, bioethanol, bioelectricity, and biochar, as well as for remediating contaminated soils and wastewater. Notable species include *Ageratum conyzoides*, *Azolla* species, *Bryophyllum pinnatum*, Jute biomass*, Chrysopogon zizanioides*, *Eichhornia crassipes, Hibiscus cannabinus*, *Ipomoea carnea*, *Pennisetum purpureum*, *Pistia stratiotes*, *Pteris vittata*, *Ricinus communis*, *Spirodela polyrhiza*, and *Xanthium strumarium*.

A study of *Ageratum conyzoides* (billy goat weed) showed its efficiency in biogas production through anaerobic digestion with cow dung [70]. Azolla species, with rapid growth potential and rich in lipids, starch, and cellulose/hemicellulose, are used for biodiesel production [71, 72]. Reliable and cost-effective biodiesel production using *Azolla filiculoides* with a graphene oxide nanocatalyst has been researched [44]. *Bryophyllum pinnatum* (cathedral bells) leaves can generate electricity in off-grid regions and produce silver nanoparticles for power generation in bioelectrochemical cells [48, 73]. Jute biomass, with high cellulose and hemicellulose content, is promising for bioethanol production [74, 75]. *Chrysopogon zizanioides* (vetiver grass) and *Eichhornia crassipes* (water hyacinth) biomass are used for bioethanol production due to their high cellulose content [51, 76]. *Hibiscus cannabinus* (kenaf) and *Ipomoea carnea* (pink morning glory) have potential for bioenergy production through their lignocellulosic biomass [53, 54, 77].

*Pennisetum purpureum* (Elephant grass) has a greater potential for second-generation bioethanol production compared to sugarcane bagasse and can generate significant electricity from biomass [55, 78]. *Pistia stratiotes* (water lettuce) can produce biogas and ethanol, although it yields less methane than other aquatic plants [56–58]. *Pteris vittata* (Chinese brake) can be converted into bio-oil, biogas, and biochar, with improved biofuel quality and reduced heavy metal content [59–61]. *Ricinus Communis* L (castor oil) is viable for sustainable biofuel in Ecuador, showing economic feasibility [62, 63]. *Spirodela polyrhiza* (duckweed) is suitable for bioethanol and biogas production due to high biomass and starch content [65–67, 79]. *Xanthium strumarium* (common cocklebur) provides a high biodiesel yield with advantageous properties [69]. It is now crucial to apply advanced technological approaches to enhance the hyperaccumulation efficiency of identified plant species and to identify any obstacles that hinder the commercialisation of the phytoremediation technique.

**Figure 1:**
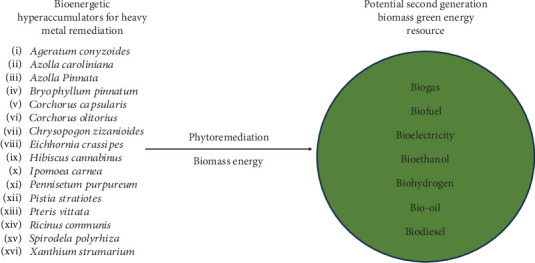
Sixteen bioenergetic hyperaccumulators for heavy metal remediation, with potential as second-generation biomass green energy resources.

Heavy metal hyperaccumulator species with bioenergy biomass potential (*n* = 16), as shown in Figure 1, have been given importance in this study due to their synergistic possibilities between phytoremediation and bioenergy production to avoid biomass disposal problems. Heavy metals cannot be metabolised, which poses potential environmental risks for hyperaccumulator species. So, there is the possibility of causing heavy metal pollution again by disposing of contaminated hyperaccumulator species. Furthermore, it is imperative to have an economically sustainable cleaning technology that is ecofriendly and capable of achieving a sustainable development goal 7 that is “affordable, reliable, sustainable and modern energy for all” and profitable by producing green energy from biomass from contaminated hyperaccumulators [80]. Furthermore, when using plant-based technology to accumulate pollutants, it is also important to consider the value of both land and time [81]. However, biomass energy can be a great alternative and sustainable source of energy for Bangladesh. By leveraging modern technology and providing institutional and financial incentives, biomass energy can contribute significantly and ensure the adaptability of Sustainable Development Goal 7—access to affordable, reliable, sustainable, and modern energy for all [82–84].

## 4. Discussion

Heavy metal pollution in both water and soil in Bangladesh arises from a combination of anthropogenic and natural sources. In water, significant contributors include industrial discharges from tanneries, mining, smelting, battery recycling, shipbreaking, and Export Processing Zones. Untreated municipal wastewater, agricultural runoff, transportation discharges, and waste from hatcheries and aquaculture farms also exacerbate contamination. Additionally, natural sources such as bedrock weathering influenced by climate change further mobilize these metals [5]. Similarly, heavy metal pollution in soil results from various sources, including mining, chemical processing, skin cleaning, shipbreaking, transportation, battery recycling, and the excessive agricultural use of pesticides and fertilizers. Notably, the tannery industry, particularly in the Hazaribagh area, significantly contributes to contamination with chemicals like chromium sulfates, dyes, and acids, introducing toxic metals such as Zn, Cd, Cr, Cu, Ni, and Pb into the environment [10]. Collectively, these pollutants pose substantial threats to the environment, agriculture, animals, and human health. Most of the metal-polluted areas are commercial and industrial bases such as tanneries, garments, dyeing, pharmaceuticals, and shipbreaking yards. Rivers, lakes, mangrove forests, and coastal areas are also polluted by heavy metals from these sources, including rapid urbanisation due to cement and metallurgical factories, and municipal and industrial wastewater [7, 14, 85, 86].

**Figure 2:**
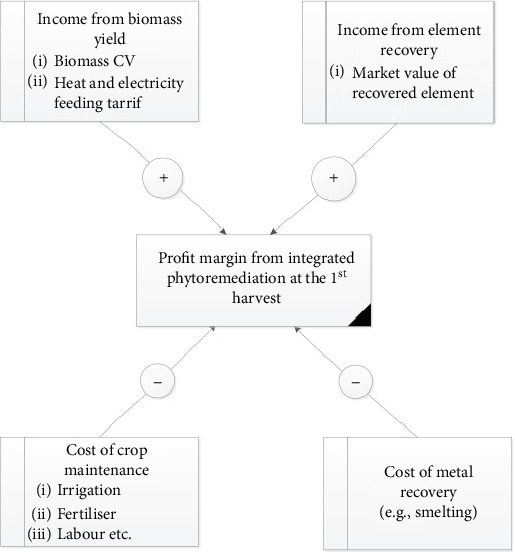
“Determinants for the economic model of integrated phytoremediation”, adapted from Jiang et al. [89].

Phytoremediation, which employs bioenergy hyperaccumulators to remove heavy metal pollutants from the environment, is a promising technology for Bangladesh. It offers potential not only for effective phytoremediation and biomass energy production but also for significant business opportunities in the region [87–90]. However, Figure 2 illustrates the key factors that impact the profitability of an integrated phytoremediation project. The model considers two direct income streams: (1) Harvested biomass, where the calorific value (CV), efficiency of the combined heat and power (CHP) unit, and heat/electricity tariffs determine profitability. Feed-in tariffs (FITs) in 63 jurisdictions provide guaranteed prices, reducing income uncertainties by eliminating energy market price fluctuations. (2) Elemental recovery from biomass, where the market value of recovered elements is considered. Costs for planting, maintaining crops, and metal recovery are deducted from the income [89].

### 4.1. Processing Biomass to Bioenergy and Its Business Potential in Bangladesh

Biomass is a conventional energy source widely used for cooking in rural areas of Bangladesh, alongside oil, natural gas, and coal. The country has a vast amount of biomass resources, which makes it a potential place for bioenergy technologies under the clean development mechanism (CDM). Bioenergy not only provides income and employment opportunities but also helps to conserve the environment. Various measures are in place to promote and produce biomass energy in Bangladesh under CDM. These measures include policy, technical, economic, and institutional measures. The goal is to encourage the creation of a bioenergy market, enhance biomass production, and link biomass producers and bioenergy utilities for a strong long-term commitment [91].

In Bangladesh, biomass currently constitutes approximately 25% of the primary energy mix, while the remaining 75% is supplied by commercial energy sources. From 2012–2013, the estimated total biomass resources available for energy purposes amounted to 90.21 million tons, with an annual energy potential equivalent to 45.91 million tons of coal. The recoverable biomass during this period possessed an energy potential of 1344.99 PJ, which corresponds to 373.71 TWh of electricity.^1^

The primary biomass resources in Bangladesh include animal dung, agricultural crop residues, municipal solid waste, and forest residues. These resources make a substantial contribution to the nation's energy production, collectively generating approximately 1574.16 PJ of energy, equivalent to 437.28 TWh of electricity. Specifically, agricultural residues contribute around 852.32 PJ, animal manure of 399.04 PJ, municipal solid waste of 112.16 PJ, and forest residues of 210.64 PJ of energy [92].

According to the national energy balance for the fiscal year 2020–2021, the primary energy supply (PES) in Bangladesh was 2,339,290.76 TJ. Approximately 27.09% of Bangladesh's PES comes from biomass, with the remaining 72.91% from commercial energy sources. Biogas facilities in dairy and poultry farms produce around 0.69 MW of electricity for cooking and energy production. Additionally, biomass gasification in the country has a capacity of 0.4 MW. However, the energy content of the generated biomass is estimated to be 386.81 TWh per year. Additionally, approximately 912.13 GWh of electricity can be produced annually from organic waste in Dhaka [93]. The government of Bangladesh aims to generate 15% of the country's total electricity from renewable sources by 2041, yet the current minimal use of renewable energy does not align with this vision [92].  Table 2 presents an overview of the biomass resources in Bangladesh and their energy potential, highlighting the contribution of various biomass types to the country's energy mix, including the energy content of generated biomass and electricity production from organic waste in Dhaka.

The use of biomass processing technology is essential for producing bioenergy, which can help reduce carbon emissions and fight against climate change. Various techniques are employed for the conversion of biomass to bioenergy, including biochemical and thermochemical methods [94]. Biochemical methods involve fermentation and enzymatic hydrolysis, while thermochemical methods include direct combustion, gasification, pyrolysis, and anaerobic digestion [95]. The approach chosen depends on the specific biomass feedstock and the desired end product.

Biotechnology and genetic engineering are crucial tools that can help enhance the production of bioenergy in the future. Different biotechnological techniques such as PCR, microarray, metagenomic, and proteomic identification can identify new microorganisms that can produce biofuels more efficiently. Similarly, genetic engineering techniques like CRISPR-Cas9, gene overexpression, metabolic engineering, and genome shuffling are playing a significant role in producing large volumes of biofuels [96].

### 4.2. Cost Information of Phytoremediation Technology

The cost and general steps for a phytoremediation project include site characterization (assessing soil and water conditions, climate, and contaminant distribution); treatability studies (investigating remediation process and rates, selecting suitable plant species, determining planting density, and finalizing site location); preliminary field testing (monitoring results on-site and adjusting design parameters as needed); full-scale remediation (implementing the remediation plan), and disposition of contaminated plant material (handling affected plant material appropriately) [97].

Over a period of 2 years, a phytoremediation project successfully removed arsenic, cadmium, and lead from contaminated soil. The total cost was $75,375.2 per hectare (or $37.7 per square meter). The initial capital and operational expenses were divided as 46.02% and 53.98%, respectively. Importantly, phytoremediation proved to be more cost-effective than other remediation methods. Furthermore, taking into account the environmental benefits, the investment pays off within 7 years [98].

However, several limitations impede the commercialisation of phytoremediation. One of the main challenges is the slow growth rate of plants. Phytoremediation is a time-consuming process that requires a long time to remove pollutants effectively. This makes it unsuitable for situations where rapid remediation is needed [99]. Additionally, only a few plant species have demonstrated effectiveness in removing pollutants from the environment, limiting the applicability of phytoremediation to specific types of pollutants and environments. Moreover, phytoremediation can be expensive due to the high cost of planting and maintaining the plants, making it challenging to compete with other cheaper remediation technologies. Finally, there is limited public awareness of phytoremediation, which makes it difficult to generate interest and investment in the technology [99].

Despite these limitations, phytoremediation has been successfully commercialised in some cases. For example, phytoremediation of organic pollutants has achieved considerable commercial success in the United States [99]. The US Environmental Protection Agency has published a *Phytoremediation Resource Guide* that lists over 100 phytoremediation overviews, field studies and demonstrations, research articles, and Internet resources [100]. The United States and China are the most frequent patent applicants for water phytoremediation technology, followed by Japan [101]. Although the development of phytoremediation into commercial technologies has been slow, it is gaining momentum for business potential [99].

Putrajaya, located in Malaysia, is an example of the integration of phytotechnology into infrastructure that increases sustainability without sacrificing continued development. It has been developed as a “garden city” and features one of the largest freshwater wetlands in the tropics, including 197 ha of wetland plants [99]. However, long-term field experiments in situ are necessary for successful soil restoration using phytoremediation [81].

### 4.3. Phytoremediation Challenges: Sustainable Solutions for Bangladesh

The phytoremediation process demands vast areas of land and consumes considerable time to complete. Furthermore, the effectiveness of phytoremediation is limited to shallow soils less than 3 ft deep and water streams located within 10 ft of the surface [102]. In Bangladesh, only a handful of plant species are suitable for phytoremediation of soils and wastewater contaminated with heavy metals. More native plant species with bioenergy potential are needed to investigate phytoremediation effectiveness. According to a study, a multifaceted approach that includes grasses, shrubs, legumes, perennial grasses, and other long-lived trees is more likely to be effective than relying on a monoculture [103].

Growing bioenergy crops with phytoremediation potential can be challenging due to several limitations that must be considered from social, economic, and environmental perspectives. One such limitation is the need to prevent biofuel contamination to avoid health hazards and environmental pollution. Additionally, achieving high-yielding biomass from bioenergetic hyperaccumulators is essential to produce maximum energy at a low cost. It is also crucial to select appropriate bioenergetic hyperaccumulator species for a given location and ensure proper implementation of the phytoremediation technique [104].

Hyperaccumulator species, due to their inability to metabolize heavy metals, pose potential environmental risks when disposing of contaminated plants, potentially causing heavy metal pollution again. Current techniques to manage plants contaminated with heavy metals after the phytoextraction process include incineration, direct disposal, ashing, and liquid extraction. Future research should focus on developing more effective, feasible, economically acceptable, and ecofriendly methods to prevent heavy metals from re-entering the soil [21].

Currently, phytoremediation research is mainly focused on three key areas. The first area involves the study of genetics, physiology, and biochemistry to enhance plant tolerance, increase biomass yield, and remove contaminants. The second area is centred on the rhizosphere processes that affect the availability of contaminants and microbes, while the third area is aimed at impacting the availability of pollutants and microbes through rhizosphere procedures [81]. Additionally, eight key areas should also be focused on to develop highly productive plant species that can accumulate heavy metals quickly in the field; eight areas are as follows: (1) plant resistance to toxic elements, (2) plant root activity, (3) absorption and short-distance transport of metal elements, (4) transformation of metals into their most mobile species, (5) translocation of metal elements through the vascular system, (6) transformation from aerial species to the best-managed element species, (7) chemical reservoirs for accumulation of toxic elements, and (8) physical reservoirs for toxic elements [105].

It is crucial to conduct strategic research on biotechnological interventions that can deploy phytoremediation technology on a field scale and make it commercially valuable. To achieve this, technology must have beneficial impacts while generating sustainable profits, which is a challenge in Bangladesh. To initiate phytoremediation technology in the country for successful economic value-added potential, several factors must be considered: (i) selecting suitable nonedible hyperaccumulator species based on the climate and soil of the contaminated area, (ii) generating value-added biomass from the chosen hyperaccumulator for land values, and (iii) improving the accumulation performance of the hyperaccumulator for time values.

Furthermore, implementing phytoremediation technology for commercial purposes can be profitable if bioenergy-sourced plants produce high-yield biomass. However, if these plants contain excessive heavy metal contaminants, they can be hazardous when disposed of. Transforming biomass into bioenergy using thermochemical methods can effectively address disposal issues for bioenergy phytoremediators. Bioenergetic hyperaccumulators offer a solution to remediate polluted environments and produce green energy resources [106]. Bioenergy is increasingly seen as the future of global energy production, with plant biomass being used to generate clean energy and reduce greenhouse gas emissions as a replacement for fossil fuels [91].

### 4.4. Strategies to Advance Phytoremediation for Sustainable Bioenergy and Environmental Cleanup in Bangladesh

**Figure 3:**
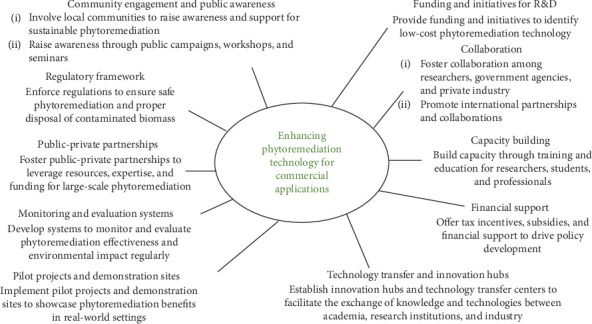
Recommendations to enhance phytoremediation technology development.

To enhance the development of phytoremediation technology from lab research to sustainable commercial applications with value-added benefits, several key recommendations, as shown in Figure 3, can be implemented. First, providing funding and initiatives for R&D to identify low-cost phytoremediation technology is essential. Establishing innovation hubs and technology transfer centres can facilitate the exchange of knowledge and technologies between academia, research institutions, and industry. For example, the Phy2Climate project, a Horizon 2020 initiative, involves partners from various countries working on soil remediation, phytoremediation, and biofuel technologies.^2^ Promoting international partnerships and sharing best practices, advanced technologies, and research findings can further support these efforts. The Phy2Climate project consortium includes 17 partners from 10 countries, fostering the exchange of knowledge and technologies.^2^ Raising awareness about the benefits of phytoremediation technology with bioenergy plants against heavy metal pollution through public campaigns, workshops, and seminars is crucial. Collaboration among researchers, government agencies, and private industry is imperative for developing and implementing phytoremediation technologies and accelerating their commercialisation using bioenergy resources. For example, the Harare City Council and Chinhoyi University of Technology (CUT) are implementing awareness campaigns to educate the local community about phytoremediation [99].

Offering tax incentives, subsidies, and other financial support to companies and institutions in the phytoremediation sector can drive the development of policies and regulations that support the implementation of phytoremediation and bioenergy technologies. Building capacity through training and education for researchers, students, and professionals is necessary to create a skilled workforce and expedite the development and implementation of bioresource technologies in Bangladesh. Additionally, developing robust monitoring and evaluation systems to assess the effectiveness and environmental impact of phytoremediation projects, implementing pilot projects and creating demonstration sites, and encouraging public-private partnerships can showcase the practical application and benefits of these technologies.

Establishing a strong regulatory framework to ensure the safe implementation and operation of phytoremediation projects, including guidelines for the disposal of contaminated biomass, and involving local communities in phytoremediation projects to raise awareness and gain support are also vital. These comprehensive recommendations are aimed at strengthening the development and commercialisation of phytoremediation technology, making it a viable and impactful solution for addressing heavy metal pollution and promoting sustainable bioenergy production in Bangladesh. Last but not least, phytoremediation with bioenergy plants is an ecofriendly approach that could successfully mitigate the heavy metal-polluted environment and contribute to affordable and clean energy production in Bangladesh.

By utilising advanced biotechnological techniques, green remediation technology has the potential to become a sustainable and commercially viable phytotechnology with bioenergy sources in the future. Gaining advanced knowledge in phytotechnology can help find solutions and overcome the limitations of phytoremediation, making it a promising technology for removing heavy metals, organic pollutants, and radionuclides from contaminated soil and water. The following overview outlines the scope and implementation of biotechnological research aimed at addressing various factors that limit the acceptance of phytoremediation technology.

Bioengineering transforming phytoremediation: Bioengineering, involving genetically modified plants, is promising for phytoremediation by removing pollutants through enhanced heavy metal uptake, translocation, and sequestration [107]. Genetic technologies can significantly improve plant health and growth. Fast-growing, high-biomass plants have been modified for better antioxidant activity, aiding heavy metal tolerance ([21, 80]. Plants have different types of proteins that help them tolerate and resist heavy metals. CRISPR/Cas9 genome editing creates transgene-free phytoremediators with increased growth, biomass, stress tolerance, and metal accumulation. Relevant genes include *ArsB*/*C*, *CopC*, *ABC*, *AtHMA4*, *PCS*, *GCS*, *HvNAS1*, *PPK*, and *vgb* [108, 109].

Rhizosphere microbes and phyto(rhizo)remediation: Rhizosphere microorganisms, such as plant growth-promoting rhizobacteria and arbuscular mycorrhizal fungi, enhance heavy metal uptake and translocation by stimulating root growth and expanding the absorptive surface area, leading to improved plant growth, metal tolerance, and heavy metal bioavailability [80]. These microbes improve phytoremediation efficiency by releasing chelating agents, acidifying soil, solubilizing phosphate, and inducing redox changes [110]. Certain heavy metal tolerant bacterial strains, like *Pseudarthrobacter oxydans* and *Pseudomonas brassicacearum*, increase the biomass, chlorophyll, carotenoid, and antioxidant enzyme activities of plants like *Sulla spinosissima* [111]. Native rhizobacteria containing plant growth regulators also enhance heavy metal tolerance, as seen in duckweed with the combination of native Pseudomonas and salicylic acid [112].

Interactions between plants and microorganisms enhance heavy metal tolerance and bioremediation through various mechanisms such as the production of siderophores, IAA, ACC deaminase, biochelators, biosurfactants, and EPS. These interactions also involve balancing ROS, phosphate solubilization, metal bioavailability, quorum sensing, and emission of VOCs [108, 113]. For instance, *Enterobacter cloacae* in transgenic canola expressed ACC deaminase to remediate arsenate-contaminated soil [114]. Additionally, CRISPR gene editing can significantly advance the investigation of plant–microbe interactions in phytoremediation [109].

Although the use of rhizobacteria is involved in the transfer and mobilisation of heavy metals, some areas need to be addressed to enhance the phytoremediation efficiency of native hyperaccumulators: (i) identification of heavy metal-tolerant rhizobacteria of the targeted (native) hyperaccumulators and (ii) investigation and elucidation of the rhizobacteria underlying specific heavy metal accumulation, distribution, and sequestration.

Nanobiotechnology, bioinformatics, and artificial intelligence (AI) in phytoremediation: Nanobiotechnology merges nanoparticles with phytoremediation, enhancing plants' ability to clean soil and water pollutants. Different nanoparticles can detoxify various contaminants, and plants naturally produce nanoparticles like nZVI to cope with environmental stress and boost growth. Nanotechnology supports phytoremediation by directly removing pollutants or promoting plant growth, showcasing significant potential. However, further research and field trials are needed for commercial success [47, 115–117].

Bioinformatics is crucial in phytoremediation research, emphasizing proteomics and bioinformatics to cost-effectively mitigate pollution. In-silico approaches help identify, express, and analyze genes related to metal tolerance, aiding genetic improvement and developing transgenic plants with enhanced phytoremediation potential. For example, the expression of genes like oligopeptide transporter 3 (OPT3) and metal-nicotianamine transporter (YSL3) under Cd stress in *Brassica oleracea* demonstrates this potential [118–120].

AI has been increasingly used in recent years to enhance and monitor the process of phytoremediation. Using AI techniques, it has become possible to make predictions about pollutant levels and optimize various process conditions to ensure the efficient removal of heavy metal contaminants [121]. AI algorithms can also be employed to analyze complex environmental data, which helps to select the right type of plants for the process, predict growth patterns, and assess the progress of pollutant removal [122, 123]. In addition, new and advanced technologies such as sensors and cameras have been used to monitor plant growth and relevant morphological parameters in the field of phytoremediation [124].

### 4.5. Bioeconomic Strategies for Phytoremediation: Optimizing Biomass Valorisation and Ecological Balance Restoration

Phytoremediation technologies hold promise for addressing environmental pollution, but they must undergo thorough screening for potential risks to human health and the environment before being put into practice. This field is interdisciplinary, combining elements from agronomy, ecological engineering, ecology, economics, and social sciences, making collaboration across various disciplines essential. Understanding how pollutants behave in plants is critical for downstream bioenergy production and for preventing the transfer of metals during biofuel production, which highlights the need to use nonedible crops. Recent global trends in phytoremediation focus on improving food production, reducing pollutants, valorising biomass, generating energy, and maintaining ecological balance. However, moving from laboratory research to practical field applications requires overcoming various complexities through collaboration among experts and the use of innovative technologies, such as nanotechnology. The challenges of sustainable development—like ensuring food security, remediating pollution, and recovering energy—are interconnected. They require precise pollution mapping and effective bioremediation strategies. Despite advances in producing bioenergy from contaminated biomass, there is still a need for specialized technical knowledge and optimization of thermochemical conversion processes. Biorefineries play a crucial role in minimizing agricultural waste and integrating biomass that has been phytoremediated into a circular bioeconomy. Long-term field studies are necessary to validate the economic feasibility of these approaches and to identify suitable plant species for sites contaminated with heavy metals. Advanced phytoremediation strategies should focus on practical application, economic viability, and sustainable development through interdisciplinary collaboration and innovative solutions [88].

The transition toward a green economy and the growing demand for low-carbon ecocities require enhancing phytoremediation's capabilities in removing contaminants, producing food, valorising biomass, generating energy, and restoring ecological balance—important factors for societal well-being and environmental protection. Achieving these goals entails extensive, large-scale, long-term field trials to thoroughly address uncontrolled variables. Currently, effective management methods to prevent contaminant transfer into the food chain are lacking, underscoring the need for appropriate bioremediation strategies, including phytoremediation and biofortification. By tackling these challenges through interdisciplinary collaboration, innovative solutions, and comprehensive life cycle assessments, we can unlock the full bioeconomic potential of phytoremediation and bioenergy production [87].

## 5. Conclusion

Bangladesh seeks to secure energy resources while addressing heavy metal contamination for sustainable development and public health. This study focuses on commercialising phytoremediation technology using bioenergetic hyperaccumulator plant species. Sixteen plant species were identified as potential bioenergy sources that are effective in accumulating heavy metals from contaminated soils and wastewater. These bioenergy plants can help address biomass disposal challenges by converting the resulting bioenergy production after the remediation process of chemical pollutants. This approach not only opens up business opportunities for phytoremediation technology but also encourages wider adoption in large-scale applications, with the aim of helping Bangladesh achieve its target of generating 15% of total electricity from renewable sources by 2041. This comprehensive review addresses the challenges and provides recommendations for transitioning phytoremediation technology using bioenergy plants from lab research to field applications. Simultaneous research and advancements in environmental and agricultural engineering can help overcome the limitations of phytoremediation technology while generating sustainable energy sources, unlocking its full bioeconomic potential. Overall, phytoremediation, as an interdisciplinary and promising bioeconomic technology for addressing environmental pollution, requires thorough risk screening, expert collaboration, innovative technologies, and effective bioremediation strategies to optimize bioenergy production and ensure sustainable development.

## Figures and Tables

**Table 1:** A list of hyperaccumulator species with the prospect of green energy production and economic value-added by-products resource in Bangladesh.

**Hyperaccumulator species investigated under laboratory or greenhouse conditions**	**English/common name**	**Potential economic value-added byproduct**	**Reference**
*Ageratum conyzoides*	Billy goat weed	Biogas production, sodium alginate film manufacture, essential oil	Paul et al. [43]
*Azolla caroliniana*	Eastern mosquito fern	Biogas, biofuel	Sathish et al. [44]
*Azolla Pinnata*	Water velvet	Biogas, biofuel	Kj et al. [45]
*Avicennia officinalis*	Indian mangrove	Natural dye (tannins), timber-wood, biofuel	Mehta et al. [46]
*Blumea lacera*	Lettuce-leaf blumea	Silver nanoparticles	Pandey et al. [47]
*Bryophyllum pinnatum*	Cathedral bells, miracle leaf, Goethe plant	Bioelectricity	Hasan and Khan [48]
*Corchorus capsularis*	Jute	Fiber, geotextile, biogas, bioethanol	Ferdous et al. [49]; Jahan [50]
*Corchorus olitorius*			
*Chrysopogon zizanioides*	Vetiver grass	Bioethanol	Neve et al. [51]
*Eichhornia crassipes*	Water hyacinth	Biogas, biofuel,	Ayanda et al. [52]
*Hibiscus cannabinus*	Kenaf	Bioethanol, biohydrogen, bioenergy	Saba et al. [53]
*Ipomoea carnea*	Pink morning glory	Bio-oil	Saikia et al. [54]
*Pennisetum purpureum*	Elephant grass	Biofuel	Danquah et al. [55]
*Pistia stratiotes*	Water lettuce	Biogas, bioethanol	Cong et al. [56]; Koley et al. [57]; Mishima et al. [58]
*Pteris vittata*	Chinese brake	Biofuel, biocomposites	Bavasso et al. [59]; Chen and Li [60]; Hu et al. [61]
*Ricinus communis*	Castor bean	Biofuel, biodiesel	Penabad Sanz et al. [62]; Vasco et al. [63]
*Schumannianthus dichotomus*	Cool mat	Handicraft-based household products	Ahmed et al. [64]
*Spirodela polyrhiza*	Duckweed	Biofuel, bioplastic, bioethanol	Cao et al. [65]; Rana et al. [66]; Romanowska-Duda et al. [67]
*Tagetes patula*	French marigold	Floriculture, biogas, commercial lutein	Chauhan et al. [68]
*Xanthium strumarium*	Common cocklebur	Biodiesel	Chutia et al. [69]

**Table 2:** Biomass resources and their energy potential in Bangladesh.

**Aspect**	**Value**
Biomass contribution to primary energy	25%
Commercial energy contribution	75%
Total biomass available (2012–2013)	90.21 million tons
Annual energy potential of biomass (2012–2013)	45.91 million tons of coal equivalent
Energy potential of recoverable biomass (2012–2013)	1344.99 PJ (equivalent to 373.71 TWh)
Agricultural residues	852.32 PJ (equivalent to 236.76 TWh)
Animal manure	399.04 PJ (equivalent to 110.84 TWh)
Municipal solid waste	112.16 PJ (equivalent to 31.15 TWh)
Forest residues	210.64 PJ (equivalent to 58.53 TWh)
Total existing biomass resources	1574.16 PJ (equivalent to 437.28 TWh)
Primary energy supply (PES) (2020–2021)	2,339,290.76 TJ
Biomass contribution to PES (2020–2021)	27.09%
Commercial energy contribution to PES (2020–2021)	72.91%
Energy content of generated biomass	386.81 TWh/year
Electricity from organic waste in Dhaka	912.13 GWh/year
Biogas plant electricity generation	Approximately 0.69 MW
Biomass gasification capacity	0.4 MW

## Data Availability

Data sharing is not applicable to this article as no new data were created or analyzed in this study.

## Endnotes

^1^Energy Scenario of Bangladesh (2022–2023) (https://hcu.portal.gov.bd/sites/default/files/files/hcu.portal.gov.bd/publications/ae775b7e_b63d_491d_81e4_b317ff8e11ca/2024-07-15-09-11-d13a3451b969fb9a3c5c74e9130c9f6c.pdf).

^2^Partners—Phy2climate (https://www.phy2climate.eu/partners/).

## Supporting Information

Supporting Information Additional supporting information can be found online in the Supporting Information section. Table S1: A list of 36 phytoremediators that are native to Bangladesh and remediate heavy metals from contaminated soil and wastewater in Bangladesh.
