# Supplementary material for: Phytoremediation: Sustainable Solutions for Heavy Metal Pollution and Bioenergy in Bangladesh
Source: ScientificWorldJournal. 2025 May 18;2025:5510989. doi: 10.1155/tswj/5510989 (PMC12124930; doi:10.1155/tswj/5510989)
Supplement: Supporting Information — Additional supporting information can be found online in the Supporting Information section. Table S1: A list of 36 phytoremediators that are native to Bangladesh and remediate heavy metals from contaminated soil and wastewater in Bangladesh. [file 5510989.f1.docx]

**Table 1S**: A list of hyperaccumulator plants that studied for the remediation of heavy metals from contaminated soil and wastewater in Bangladesh

| **Hyperaccumulator species in study** | **Source of pollutant to conduct experiment** | | **Accumulated heavy metals** | | **Translocation** | **Ref** |
| --- | --- | --- | --- | --- | --- | --- |
| *Acanthus ilicifolius* L. | Ship breaking area of Sitakunda coast | | Cu, Zn, As, Fe | | Root and shoot | Rahman et al., 2019 |
| *Ageratum conyzoides* L. | Arsenic contaminated (underground water) areas of four districts (Khulna, Satkhira, Bagerhat, and Brahmanbaria) | | Ar | | Root and top (stem, leaves, flowers, and seeds) | Mahmud et al., 2008) |
| *Blumea lacera* (Burm.f.) DC. |  |  |  |  |  |  |
| *Clerodendrum trichotomum* Thunb. |  |  |  |  |  |  |
| *Mikania cordata* (Burm.f.) B.L.Rob. |  |  |  |  |  |  |
| *Ricinus communis* L. |  |  |  |  |  |  |
| *Dryopteris filix-mas* (L.) Schott |  |  |  |  |  |  |
| *Corchorus capsularis* L. | Arsenic contaminated soil of Mymensingh  District; industrially  polluted region of Bhaluka sub-district | | Ar; Pb | | Root and shoot | Uddin Nizam et al., 2016; Nizam Uddin, 2016 |
| *Hibiscus cannabinus* L. |  |  |  |  |  |  |
| *Hibiscus sabdariffa* L. |  |  |  |  |  |  |
| *Bryophyllum pinnatum* (Lam.) Oken | Textile industries area in Kaliakair | | Cu and Zn | | Root, stem, and leaf | Riza and Hoque, 2021 |
| *Brassica juncea* (L.) Czern. | Uppermost soil layer of  Buriganga riverside;  Sodium arsenic (NaAsO_2_) in Hoagland solution; Buriganga riverbed sediments | | Cd, Ar, Cr, Pb, Cu, and Zn | | Root, shoot, leaf, and fruit | Choudhury et al., 2016; Halder and Anirban, 2023; Rahman et al., 2018 |
| *Tagetes patula* L. |  |  |  |  |  |  |
| *Avicennia Officinalis* L. | Mongla industrial area | | Sr, Mn, Zn, Cu, Fe | | Root and leaf | Hossain et al., 2022 |
| *Excoecaria agallocha* L. |  |  |  |  |  |  |
| *Sonneratia apetala* Buch.-Ham. |  |  |  |  |  |  |
| *Xanthium strumarium* Lour. | Water, soil and sediments of the wastewater discharge channel of Savar tannery industrial estate on the bank of the Dhaleshwari River | | Cr | | Root | Hasan et al., 2021 |
| *Pteris vittata* L. | Arsenic trioxide (As_2_O_3_), Disodium arsenate (Na_2_HAsO_4_) contaminated soil | | Ar | | Root to shoot | Islam et al., 2010; Mayda et al., 2014 |
| *Pennisetum purpureum* Schumach. | Tannery sludge | | Cr, Zn, Cu, Pb | | Root, shoot and leaf | Juel et al., 2021 |
| Salt marsh, *Porteresia sp.* | Sediments of coastal areas | | Mn and Pb | | Root, shoot and leaf | Hossain et al., 2021 |
| *Echinochloa crus-galli* (L.) P.Beauv. | Naturally and artificially arsenic (Sodium arsenate, NaAsO_2_) contaminated soil;  Industrially polluted heavy  metal-contaminated topsoil;  Disodium hydrogen arsenate heptahydrate (Na_2_HAsO_4_.7H_2_O) contaminated soil;  Boron contaminated soil of rice field for ***E. crus-galli*** | | Ar, Pb, and Cr, Br | | Root and shoot | Islam et al., 2013; Shaheen et al., 2007; Sultana and Kobayashi, 2011; Sultana et al., 2022 |
| *Eichhornia crassipes* (Mart.) Solms |  |  |  |  |  |  |
| *Monochoria hastata* (L.) Solms |  |  |  |  |  |  |
| *Spirodela polyrhiza* (L.) Schleid. | Grown in three test concentrations of  arsenate and dimethylarsinic acid (DMAA) | | Ar | | Root | Rahman et al., 2007 |
| *Fimbristylis miliacea* (L.) Vahl | Boron Contaminated soil of rice field | | Br | |  | Shaheen et al., 2007 |
| *Alternanthera philoxeroides* (Mart.) Griseb. |  |  |  |  |  |  |
| *Chrysopogon zizanioides* (L.) Roberty | Arsenic contaminated soil | | Ar | | Root and leaf | Islam et al., 2016 |
| *Ipomoea carnea* Jacq. | Contaminated soil | | Cr and Ar | |  | Sarker et al., 2024 |
| CVL-1 (*Corchorus capsularis* L.) | Sodium meta-arsenite (NaAsO_2_) contaminated soil in the green house | | Ar | |  | Nahar et al., 2022 |
| OM-1 (*Corchorus*  *Olitorius* L.) |  |  |  |  |  |  |
| **Studied hyperaccumulators of heavy metals from wastewater** | | | | | | |
| *Eichhornia crassipes* (Mart.) Solms | | Arsenic contaminated (underground water) areas of (Khulna, Satkhira, Bagerhat, Brahmanbaria);  Pharmaceutical wastewater (for *E. crassipes*) | | Ar; Cr and Ni | | Das et al., 2023; Mahmud et al., 2008 |
| *Spirodela polyrhiza* (L.) Schleid. | |  |  |  |  |  |
| *Azolla pinnata* R.Br. | |  |  |  |  |  |
| *Monochoria vaginalis* C.Presl | |  |  |  |  |  |
| *Azolla caroliniana* Willd. | | Water from Polluted Hatirjheel lake, Uttara Lake, and Turag River of Dhaka City | | Remediate eutrophic water and improve physiochemical properties of water | | Nahar, 2020 |
| *Pistia stratiotes* L. | | Pollutant load of eutrophic water from four lakes (Uttara, Dhanmondi, Gulshan, and Hatirjheel); Pharmaceutical wastewater of Radiant Pharmaceuticals Limited, Dhaka | | remediate eutrophic water and improve physiochemical properties of water;  Cr and Ni | | Das et al., 2023; Nahar and Hoque, 2021 |
| *Schumannianthus dichotomus* (Kuntze) Veldkamp & I.M.Turner | | pollutants in a lab-scale vertical subsurface flow constructed wetland | | Biochemical pollutants | | Rahman et al., 2022 |

Ar=Arsenic, Br=Boron, Cd=Cadmium, Cr=Chromium, Cu=Copper, Fe=Iron, Mn= Manganese, Ni=Nickel, Pb=lead, Sr= Strontium, Zn=Zinc.

**References**

Choudhury, M.R., M.S. Islam, Z.U. Ahmed, and F. Nayar (2016) Phytoremediation of heavy metal contaminated Buriganga riverbed sediment by Indian mustard and marigold plants. *Env Prog and Sustain Energy* 35: 117–124.

Das, E.J., Md.A.R. Bhuiyan, and Md.M. Hasan (2023) Implementation of water hyacinth (*Eichhornia crassipes*) and water lettuce (Pistia stratiotes) in the re-treatment of conventionally treated pharmaceutical wastewater: a case study of Radiant Pharmaceuticals Limited, Dhaka, Bangladesh. *Environ Monit Assess* 195: 1210.

Halder, S., and A. Anirban (2023) Phytoremediation of EMS Induced *Brassica Juncea* Heavy Metal Hyperaccumulator Genotypes. SSRN.

Hasan, S.Md.M., Md.A. Akber, Md.M. Bahar, Md.A. Islam, Md.A. Akbor, Md.A.B. Siddique, and Md.A. Islam (2021) Chromium Contamination from Tanning Industries and Phytoremediation Potential of Native Plants: A Study of Savar Tannery Industrial Estate in Dhaka, Bangladesh. *Bull Environ Contam Toxicol* 106: 1024–1032.

Hossain, M.B., Md.R.J. Rakib, Y.N. Jolly, and M. Rahman (2021) Metals uptake and translocation in salt marsh macrophytes, Porteresia sp. from Bangladesh coastal area. *Science of The Total Environment* 764: 144637.

Hossain, M.B., Z. Masum, M.S. Rahman, J. Yu, Md.A. Noman, Y.N. Jolly, B.A. Begum, B.A. Paray, and T. Arai (2022) Heavy Metal Accumulation and Phytoremediation Potentiality of Some Selected Mangrove Species from the World’s Largest Mangrove Forest. *Biology* 11: 1144.

Islam, J.M., B. Kim, N. Laiju, T. Nasirullah, and M.N. Miah (2010) Arsenic Concentrations of Groundwater and Rice Grains in Bangladesh and Phytoremediation. *Journal of Korean Society on Water Quality* 26: 116–124.

Islam, Md.S., Md. Wahid-Uz-Zaman, and Md.M. Rahman (2013) Phytoaccumulation of Arsenic from Arsenic Contaminated Soils by *Eichhornia Crassipes* L.,*Echinochloa Crusgalli* L. and *Monochoria Hastata* L. in Bangladesh. *International Journal of Environmental Protection* 3: 17–27.

Islam, M.S., A.B. Siddique, F. Islam, and S. Mallick (2016) Phytoremediation of Arsenic Contaminated Soil by Vegetation . in *BUET-ANWAR ISPAT 1st Bangladesh Civil Engineering SUMMIT*, BUET, Dhaka, Bangladesh.

Juel, M.A.I., T.K. Dey, M.I.S. Akash, and K.K. Das (2021) Heavy Metals Phytoremediation Potential of Napier Grass Cultivated on Tannery Sludge in Bangladesh. *J. of Eng. Sci.* 12: 35–41.

Mahmud, R., N. Inoue, S. Kasajima, and R. Shaheen (2008) Assessment of Potential Indigenous Plant Species for the Phytoremediation of Arsenic-Contaminated Areas of Bangladesh. *International Journal of Phytoremediation* 10: 119–132.

Mayda, U., M.A. Akond, M.I. Manirul, H. Mehraj, and A.J. Uddin (2014) MITIGATION OF SOIL ARSENIC BY PHYTOREMEDIATION PROCESS. *International Journal of Business, Social and Scientific Research* 1: 87–94.

Nahar, K. (2020) Azolla (Caroliniana): An Aquatic Energy Crop for Remediation of Eutrophic Ecosystems with Prospect of Biofuel Production in Bangladesh. *Asia Pac. j. energy environ.* 7: 79–XX.

Nahar, K., and S. Hoque (2021) Phytoremediation to improve eutrophic ecosystem by the floating aquatic macrophyte, water lettuce (Pistia stratiotes L.) at lab scale. *The Egyptian Journal of Aquatic Research* 47: 231–237.

Nahar, N., M.N. Gani, and S.I. Huq (2022) Investigating the Potential Response of Jute Varieties for Phytoremediation of Arsenic Contaminated Soil. *Pollution* 8.

Nizam Uddin, Md. (2016) Phytoremediation Potentiality of Lead from Contaminated Soils by Fibrous Crop Varieties. *AJASR* 2: 22.

Rahman, M.A., H. Hasegawa, K. Ueda, T. Maki, C. Okumura, and M.M. Rahman (2007) Arsenic accumulation in duckweed (Spirodela polyrhiza L.): A good option for phytoremediation. *Chemosphere* 69: 493–499.

Rahman, M., Md. Jakariya, N. Haq, and M.A. Islam (2018) Prospect of phytoaccumulation of arsenic by *Brassica juncea* (*L.*) in Bangladesh. *International Journal of Phytoremediation* 20: 1025–1032.

Rahman, M.S., M.B. Hossain, S.M.O.F. Babu, M. Rahman, A.S.S. Ahmed, Y.N. Jolly, T.R. Choudhury, B.A. Begum, J. Kabir, and S. Akter (2019) Source of metal contamination in sediment, their ecological risk, and phytoremediation ability of the studied mangrove plants in ship breaking area, Bangladesh. *Marine Pollution Bulletin* 141: 137–146.

Rahman, M.-A., M.-H. Rahaman, S. Yasmeen, M.M. Rahman, F.M. Rabbi, O.R. Shuvo, and Usamah (2022) Phytoremediation potential of *Schumannianthus dichotomus* in vertical subsurface flow constructed wetland. *Environmental Challenges* 9: 100631.

Riza, M., and S. Hoque (2021) Phytoremediation of Copper and Zinc Contaminated Soil around Textile Industries using *Bryophyllum pinnatum* Plant. *J. Ecol. Eng.* 22: 88–97.

Sarker, S.S., S. Akter, M.A.B. Siddique, K.M.J. Rahman, S. Nahar, and S.A. Sharmin (2024) Chromium and arsenic bioaccumulation and biomass potential of pink morning glory (*Ipomoea carnea* Jacq.). *Environ Sci Pollut Res Int* 31: 2187–2197.

SHAHEEN, R., M.T. AREFIN, and R. MAHMUD (2007) PHYTOREMEDIATION OF BORON CONTAMINATED SOILS BY NATURALLY GROWN WEEDS. *J. Soil. Nature* 1: 1–31.

Sultana, R., and K. Kobayashi (2011) Potential of barnyard grass to remediate arsenic‐contaminated soil. *Weed Biology and Management* 11: 12–17.

Sultana, R., T. Ahmed, S.M.N. Islam, and M.N. Uddin (2022) Barnyard grass (Echinochloa crus-galli L.) as a candidate plant for phytoremediation of arsenic from arsenic-amended and industrially polluted soils. *Front. Soil Sci.* 2: 927589.

Uddin Nizam, M., M. Wahid-U-Zzaman, M. Mokhlesur Rahman, and J.-E. Kim (2016) Phytoremediation Potential of Kenaf (Hibiscus cannabinus L.), Mesta (Hibiscus sabdariffa L.), and Jute (Corchorus capsularis L.) in Arsenic-contaminated Soil. *Korean Journal of Environmental Agriculture* 35: 111–120.
